# Instrumental swallowing studies for the prevention of pulmonary morbidity in children and the importance of multi-disciplinary teams^☆^

**DOI:** 10.1016/j.jped.2024.05.002

**Published:** 2024-05-29

**Authors:** R. Paul Boesch

**Affiliations:** Mayo Clinic, Division of Pediatric Pulmonology, Rochester, MN, USA

Due to the increasing survivorship of children born with severe prematurity and complex conditions, the prevalence of dysphagia is increasing. Dysphagia is highly prevalent amongst children with multisystem disease, especially central neurological disease, but also occurs in children without risk factors.^1^, ^2^, ^3^, ^4^, ^5^, ^6^ Symptoms associated with chronic aspiration are non-specific, necessitating a high index of suspicion.^7^^,^^8^ Despite the known consequences of lung injury from aspiration, episodes of pneumonia are not frequent or reliably predictive of aspiration or morbidity such as bronchiectasis.^8^, ^9^, ^10^ Silent aspiration is particularly common amongst infants and toddlers, children with laryngeal clefts, and those with neurological disease, further resulting in greater risk for lung injury prior to identification and management.^8^^,^^11^^,^^12^ Given that intervention can stabilize or reverse early lung injury, prevention of long-term pulmonary disease is an important focus.

In this issue, Pazinatto et al. report a cohort of 60 children referred for evaluation of suspected dysphagia. Similar to previous studies, they identified that while there is a correlation between assessment made by clinical feeding evaluations (CFE) and fiberoptic endoscopic evaluations of swallowing (FEES), CFE failed to identify 20% of patients with significant aspiration on FEES.^13^ This confirms the importance of the addition of an instrumental swallow study, such as FEES, to a CFE in the evaluation of a child at risk for aspiration. What is also notable in this study is the length of time between symptom onset and evaluation (Median 18 months and 70% > 1 year). The time to assessment, very high rate of neurologic disease, pulmonary morbidity, and very high prevalence of severe dysphagia suggest an underutilization of this important resource. As the authors note, this may be attributed to overarching medical system limitations such as the availability of specifically trained pediatric otolaryngologists and speech-language pathologists, an overloaded medical system, established referral and management patterns, and appropriate awareness amongst potential referring providers. These potential referral delays are even more relevant given the reported impact on the care plan such an evaluation allows.

The importance of timely evaluation and management cannot be overstated. In one study, amongst a cohort of children with an instrumental swallow study positive for aspiration, two-thirds were found to have bronchiectasis, even as early as 8 months of age.^9^ In this same cohort, nearly all children with central nervous system disease and aspiration had bronchiectasis, likely due to the co-existence of effective airway clearance that is often present in such patients. Development of bronchiectasis indicates that there has been sufficient insult to airways to result in injury. Such injury can become permanent and progressive and adds additional lifelong morbidity and mortality, but, if identified and managed early, can stabilize or even resolve. It is also notable that there were no clinical features that predicted the presence of bronchiectasis (i.e., worse aspiration) and 97% of aspirating children had clear chest findings on auscultation.^9^

The symptoms associated with aspiration are non-specific and include chronic cough, congestion, wheezing, recurrent respiratory infections, failure to thrive, or simply a wet vocal quality. Respiratory symptoms may present or worsen around feeding but in children with CNS disease, laryngeal clefts, and in children under age 3, aspiration is silent up to 43–89% of the time.^9^^,^^12^^,^^13^ While aspiration is more common amongst children with other risk factors, such as prematurity, congenital heart disease, CNS disease, craniofacial abnormalities, certain genetic syndromes, and anatomic or functional abnormalities of the aerodigestive tract, there is a sizeable cohort of infants and young children with chronic aspiration without any identifiable risk factors.^5^^,^^6^ For this reason, the possibility of chronic aspiration should be considered for many children with chronic respiratory symptoms, especially in infancy and toddlerhood. It is important to re-state that recurrent pneumonia is a presenting feature in only 0–41% of children with aspiration.^9^, ^10^, ^11^ This complicates timely referral for evaluation of potential swallowing dysfunction and aspiration, often until after lung injury has developed unless an appropriate index of suspicion is maintained.

The primary organ of damage from aspiration is the lungs and the primary pathology is that of chronic bronchiolar inflammation and injury with expansion of bronchial-associated lymphatic tissue, leading to bronchiectasis, primarily in a dependent distribution. Animal models of chronic small-volume aspiration demonstrate this pattern. Particularly notable in one such model was the finding that lung injury was prominent in rats who were made to repeatedly aspirate very small volumes of whole gastric fluid, non-acidified gastric fluid, and to a slightly lesser degree ground food particles, but no injury at all was detectable in those aspirating small volumes of thin fluid of pH 2.2.^14^ This highlights the particular damage from aspiration of particulates in gastric fluid, food, and even thicker liquids, but not acid itself. Another animal study documented more lung disease in rats aspirating liquids thickened with commercial thickener than those aspirating thin liquids.^15^ Furthermore, it has also been shown that the frequency of significant respiratory infections is similar amongst those found with penetration alone (as compared to aspiration) and both exceed what is experienced in children with normal swallowing.^16^ Lastly is the importance of the opportunity to practice swallowing in order to improve swallow function over time. Children who are managed with complete exclusion of oral feeding (even water) are far less likely to improve their swallowing skills over time.^17^ Taken together, this evidence calls attention to the importance of having the consistent presence of a speech-language pathologist with specific expertise in pediatric swallowing disorders on teams evaluating and managing children suspected of aspiration. Such skilled providers are required for accurate assessment of swallowing dysfunction in the context of complex clinical status as well as for the development of individually tailored feeding plans, therapy, and ongoing monitoring and adjustment.

The authors are commended for describing the feasibility and findings of FEES in their pediatric cohort in Brazil. As they state, there is a need for expanded availability and utilization of such studies in order to identify aspirating children and prevent potential lifelong pulmonary morbidity. The evaluation of and management of suspected dysphagia is even further aided by a coordinated and engaged team, including gastroenterologists and pulmonologists, as exemplified by the aerodigestive model of care.^18^^,^^19^ Developing such coordinated practices for this population has been well proven to improve timely and accurate diagnosis, reduce hospital stays, lower cost of care, decrease risk, improve operating room utilization, and be financially feasible with high caregiver satisfaction in the United States healthcare structure.^20^, ^21^, ^22^, ^23^, ^24^, ^25^, ^26^, ^27^, ^28^, ^29^ Development of such multidisciplinary teams also establishes a framework for the multi-disciplinary evaluation of other complex pediatric patients. Examples of such aerodigestive programs currently exist in South America, but only in a few centers, and there are substantial barriers to the creation and maintenance of these valuable programs. Given that pediatric dysphagia with aspiration is common and associated with significant respiratory morbidity, it will be important and impactful to further develop multi-disciplinary aerodigestive programs in other regions on the continent and to support the stability and expansion of those currently in existence.

## Conflicts of interest

The author declares no conflicts of interest
